# Hydration behaviors of nonfouling zwitterionic materials†

**DOI:** 10.1039/d3sc01977b

**Published:** 2023-06-06

**Authors:** Pranab Sarker, Tieyi Lu, Di Liu, Guangyao Wu, Hanning Chen, Md Symon Jahan Sajib, Shaoyi Jiang, Zhan Chen, Tao Wei

**Affiliations:** a Department of Chemical Engineering, Howard University Washington D.C. USA tao.wei@howard.edu; b Department of Chemistry, University of Michigan Ann Arbor Michigan USA zhanc@umich.edu; c Meinig School of Biomedical Engineering, Cornell University Ithaca NY 14853 USA sj19@cornell.edu; d Texas Advanced Computing Center, The University of Texas at Austin Austin Texas USA

## Abstract

Zwitterionic materials have emerged as highly effective ultralow fouling materials for many applications, however the underlying mechanism of fouling resistance remains unclear. Using *ab initio* molecular dynamics simulations and surface-sensitive sum frequency generation vibrational spectroscopy, we studied the hydration behaviors of zwitterionic materials, including trimethylamine-*N*-oxide (TMAO) and carboxybetaines of different charge-separation distances, to understand their fouling-resistant mechanism and provide a design principle for improved performance. Our study reveals that the interplay among hydrogen bonding, net charge, and dipole moment is crucial to the fouling-resistant capabilities of zwitterionic materials. Shortening of the zwitterionic spacing strengthens hydrogen bonding with water against biomolecule attachment due to the increased electrostatic and induction interactions, charge transfer, and improved structural stability. Moreover, the shortened charge separation reduces the dipole moment of zwitterionic materials with an intrinsic near-neutral net charge, decreasing their electrostatic and dipole–dipole interactions with biofoulers, and increasing their resistance to fouling. Compared to carboxybetaine compounds, TMAO has the shortest zwitterionic spacing and exhibits the strongest hydrogen bonding, the smallest net charge, and the minimum dipole moment, making it an excellent nonfouling material.

## Abstract 2

Understanding the nonfouling mechanism of zwitterionic materials is crucial for their broad applications. Our study reveals the importance of strong hydration bonding, near-neutral net charge, and small dipole moment in achieving fouling resistance.

## Graphical abstract

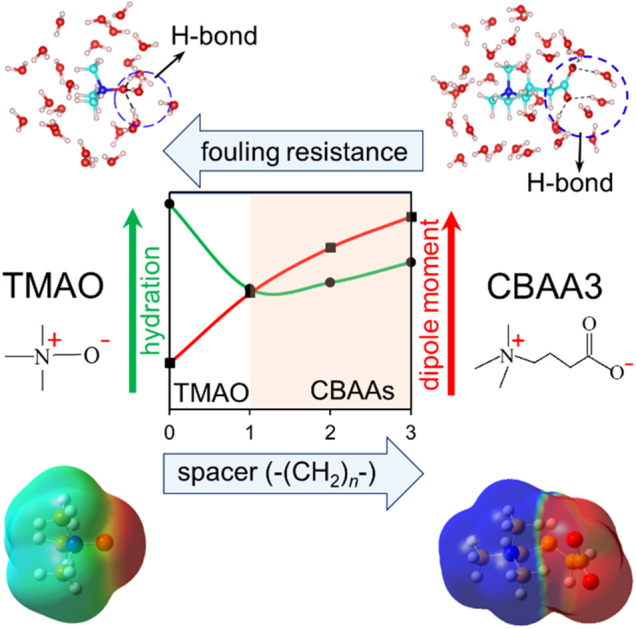

## Introduction

Biofouling is a common phenomenon that results from the attachment of biomolecules and microorganisms like proteins, cells, bacteria, mussels, algae, and seaweed to submerged surfaces, resulting in negative impacts on various applications.^1,2^ For instance, in biomedical applications, the nonspecific attachment of proteins and cells to biomaterials after implantation can trigger adverse immune responses, leading to an irrevocable foreign body reaction.^3^ In the marine industry, biofouling can increase the hydrodynamic drag of vessels and cause pitting and crevice corrosion of metal surfaces.^4,5^ Marine biofouling-resistant coatings have traditionally relied on toxic biocide-containing paints, such as those containing tributyl tin-, copper-, and zinc-based biocides.^6,7^ There is a high demand for novel environmentally benign or biocompatible coating materials that are effective against biofouling.^8^

Even a small amount of protein adsorption (<5 ng cm^−2^) on a surface can trigger biofouling propagation by promoting the attachment and growth of later-biofoulers.^9,10^ Effective fouling-resistant coatings can prevent protein attachment and biofouling propagation.^9,11,12^ Strong surface hydration was found to act as a barrier against biomolecules, preventing them from being adsorbed on submerged surfaces.^11,12^ Hence, strong surface hydration is generally regarded as a crucial factor in the design of effective fouling-resistant coating materials.^4,11,13^

Zwitterionic materials, which contain equally charged positive and negative moieties, have emerged as highly effective ultralow fouling materials for biomedical and engineering applications.^11,12,14–18^ Moreover, these materials are non-toxic, biocompatible, chemically stable, easy to prepare, and low-cost.^14,19^ Although zwitterionic materials demonstrate better salt-resistant properties compared to polyethylene glycol, their hydration can still be compromised by salt molecules,^19^ when used in high-salt environments such as blood and seawater. To overcome this issue, trimethylamine-*N*-oxide (TMAO) containing polymer, which exhibits excellent fouling-resistant activity in high-salt solutions, has been recently developed.^11,12^ The direct connection between the positive and negative moieties, *i.e.*, a short distance with no atom in between, in TMAO is believed to be the critical factor for its outstanding fouling-resistant property. Experimental observations^14,20^ rationalized that the fouling-resistant property of zwitterionic materials increases as the length of the zwitterionic spacing (*i.e.*, the separation between positively and negatively charged moieties) decreases. However, why and how the spacer controls the hydration and fouling resistance of zwitterionic materials is not fully understood, warranting a comprehensive investigation to this end.

**Scheme 1:**
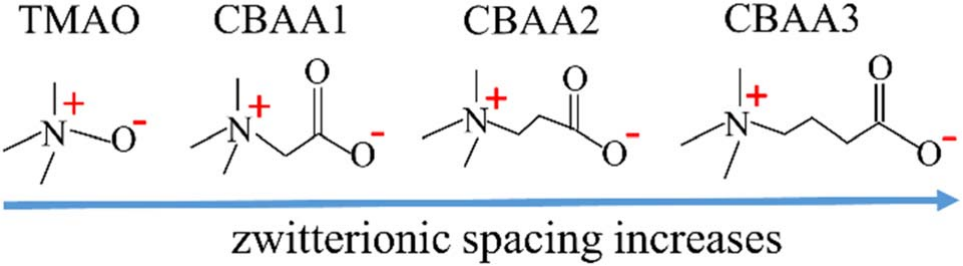
Molecular structures of zwitterions with varying zwitterionic spacing between oppositely-charged moieties.

In this study, we provide an in-depth understanding of the zwitterionic materials' hydration behavior that contributes to the fouling-resistant properties. We employed *ab initio* molecular dynamics (AIMD) simulations at the quantum scale and the experiment of surface-sensitive sum frequency generation (SFG) vibrational spectroscopy to investigate four zwitterionic materials: TMAO and CBAA1, CBAA2, and CBAA3 (*i.e.*, –(CH_2_)_*n*_–, *n* = 0, 1, 2, 3) (Scheme 1). These four materials have varying spaces between the negative and positive charges, following the trend of TMAO < CBAA1 < CBAA2 < CBAA3. Our study elucidates that the hydration behavior (hydration bonding (HB), net charge, and dipole moment) is key to the fouling-resistant capability of zwitterionic materials. We analyzed zwitterion–water interactions and the structural stability of solvated zwitterions to determine the enthalpy and conformational entropy effects on hydration. We also estimated the net charge of solvated zwitterions by taking into account the effect of charge transfer between zwitterions and water. Furthermore, we investigated the molecular dipole moment of zwitterions to explain their electrostatic and dipole–dipole interactions with biofoulants.

As will be discussed, hydration strength is insufficient to rationalize their fouling-resistant activities observed in the experiment.^11,20^ The other two factors of net charge and dipole moment need to be considered to evaluate the fouling-resistant activities of materials. When the space between the negatively and positively charged moieties of a zwitterion is short, as in TMAO (which has a single chemical bond in between), strong surface hydration, favorable zwitterion–water interactions, stable structure, small net charge, and small dipole moment contribute to its excellent character in fouling-resistance. For CBAA1 and CBAA2, which have longer charge separation spaces than TMAO, a similar hydration behavior on the entire molecular surface and zwitterion–water interactions were observed. However, these two compounds differ in their hydrogen bonding strengths, structural stabilities, and dipole moment. CBAA1, with its more stable structure and smaller dipole moment, exhibits better fouling-resistant activity than CBAA2. When the space between the negative and positive charges is too long, as in the case of CBAA3, despite having stronger surface hydration and more favorable water interaction, the fouling-resistant activity of CBAA3 is lower than that of CBAA1 and CBAA2.^20^ This is because when the space is too long, the dipole moment and surface electrostatic potential of a zwitterionic compound are also large. They may lead to strong attractive electrostatic and dipole–dipole interactions with other molecules or media, resulting in biofouling. More specifically, we answer why a shorter distance between the oppositely charged groups enhances the fouling-resistant activity of zwitterionic compounds, revealing a fundamentally important design principle to develop nonfouling zwitterionic materials with improved properties.

## Methods

### Quantum and atomistic simulations

The DFT (density functional theory)-based Car-Parrinello AIMD^21^ implemented in the Quantum Espresso^22^ (version 6.3) was used to study the hydration of zwitterions. Each zwitterion was solvated with 119 water molecules in a cubic box with a size of 16.2 Å and in periodic boundary conditions. Prior to equilibration in AIMD, a system underwent re-relaxation with atomistic MD, following our previous work.^11^ Atomistic molecular dynamics (MD) simulations were performed using the GROMACS software^23^ (version 2019.6) with CHARMM36 forcefield^24^ and TIP3P water model following our previous paper.^11^ Partial charges were estimated using quantum simulations and the restrained electrostatic potential method,^25^ as detailed in the ESI.† For the AIMD, we used the norm-conserving pseudopotentials (of Troullier–Martins form for C, N, O^26^ and of Von Barth-Car for H,^27^ respectively) and BYLP-parameterized generalized gradient approximation (BYLP-GGA^28,29^) and empirical Grimme DFT-D2 method^30^ for the van der Waals corrections for all AIMD calculations. The fictitious mass (*μ*) for electrons was set at *μ* = 300 a.u. for all zwitterions except for CBAA2 (*μ* = 400 a.u.), with a mass cut-off of 2.5 Ry, and a time step of 5 a.u. (0.121 fs). The thermostat frequencies were 10 THz, 20 THz, 20 THz, and 10 THz for TMAO, CBAA1, CBAA2, and CBAA3, respectively, estimated from the fluctuations of potential energies of their *NVE* ensemble. All systems were then gradually heated up from 20 K to 298.15 K, followed by a relaxation for over 100 ps at 298.15 K in the *NVT ensemble*. Further relaxations were carried out at 298.15 K for 200 ps and 280 ps for TMAO and CBAAs, respectively, in the NPT to achieve equilibrium.

### Simulation data analysis

The symmetry-adapted perturbation theory (SAPT)^31^ implemented in the Psi4 software^32^ (version 1.3) was used to study the non-covalent interaction (electrostatic, exchange, induction, and dispersion)^31,33^ between a zwitterion and water molecules within the first hydration shell. About 30 zwitterion–water complexes in the first hydration shell were used for the HB analysis, while about 10 configurations were used for analyzing non-hydrogen-bonded (nonHB) interactions to reduce the computational cost for large zwitterion–nonHB–water complexes. Both HB- and nonHB-interactions were estimated with the lowest order scaled-SAPT at the Hartree–Fock (HF) (sSAPT0-HF^34–36^) level, with the aug-cc-pvdz basis set. The charge transfer energy^37,38^ related to the HB between water hydrogen and zwitterionic oxygen was calculated. Using the same basis set, we also calculated the dipole moment of zwitterions at the HF level with Psi4.^32^ Note that the molecular dipole moment is the sum of the electronic and nuclear dipole moments. In Psi4,^32^ the electronic dipole moment is estimated upon solving the one-electron integrals in the Obara–Saika recursive scheme,^39^ whereas the nuclear counterpart is calculated by summing the contributions of all atomic nuclei.

To analyze HB strength and net charge of zwitterion, the quantum theory of atoms in molecules (QTAIM)^40,41^ framework was employed for the zwitterion–HB-water complexes and zwitterions with total 119 solvation water molecules, respectively, using the same configurations in the SAPT analysis. For small zwitterion–HB-water complexes, we computed their charge densities using Gaussian16 software^42^ with the hybrid functional M062X and the 6-311++G(d,p) basis,^43^ and analyzed the HB strength in Fig. 4d (right) from the ratio between kinetic and potential energy densities (−*G*(*r*)/*V*(*r*)) at the bond critical point between zwitterionic oxygen and water hydrogen using the Multiwfn software.^44^ For the large systems of zwitterions with 119 water molecules, we computed the charge density using Quantum Espresso with the same pseudopotentials, cut-offs, and plane-wave basis set as used in the AIMD, but with the PBE-GGA^45–47^ functional and DFT-D3 (ref. 48) van der Waals corrections. Subsequently, we computed Bader atomic charges utilizing those charge densities with the Bader charge analysis code.^49^

The conformational entropy *S* of each zwitterion is calculated with GROMACS^23^ (version 2019.6) using AIMD configurations and the quasi-harmonic approximation theory^50^
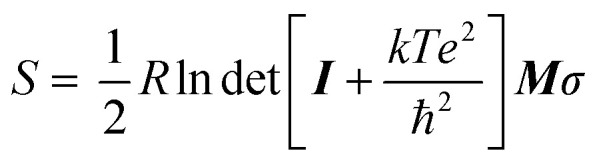
where *R* is the gas constant, ‘det’ denotes the matrix determinant, ***I*** is the identity matrix, *k* is the Boltzmann constant, *T* is the temperature, *e* is the Euler's number, ℏ is the reduced Planck constant, ***M*** is the mass matrix, and *σ* is the covariance matrix of the Cartesian coordinates.

### Sum frequency generation (SFG) vibrational spectroscopy

**Scheme 2:**
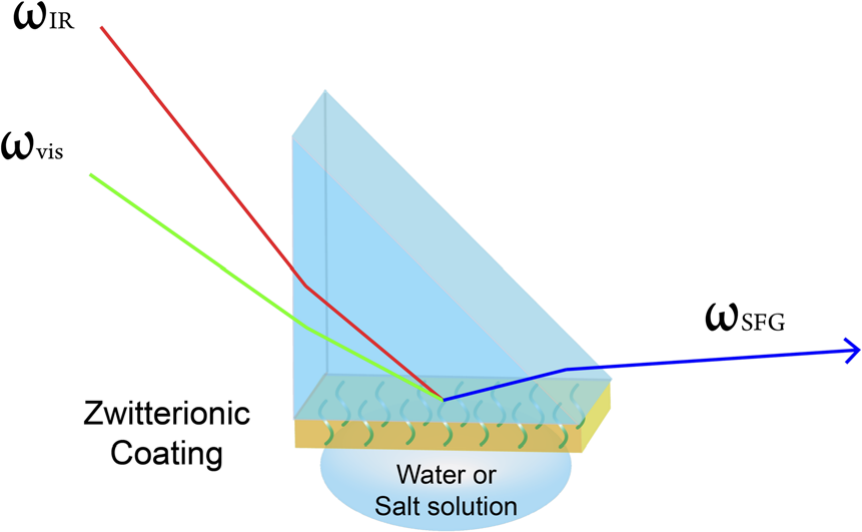
Sample geometry used to collect SFG spectra from zwitterionic polymer/water or polymer/salt solution interfaces.

**Fig. 1:**
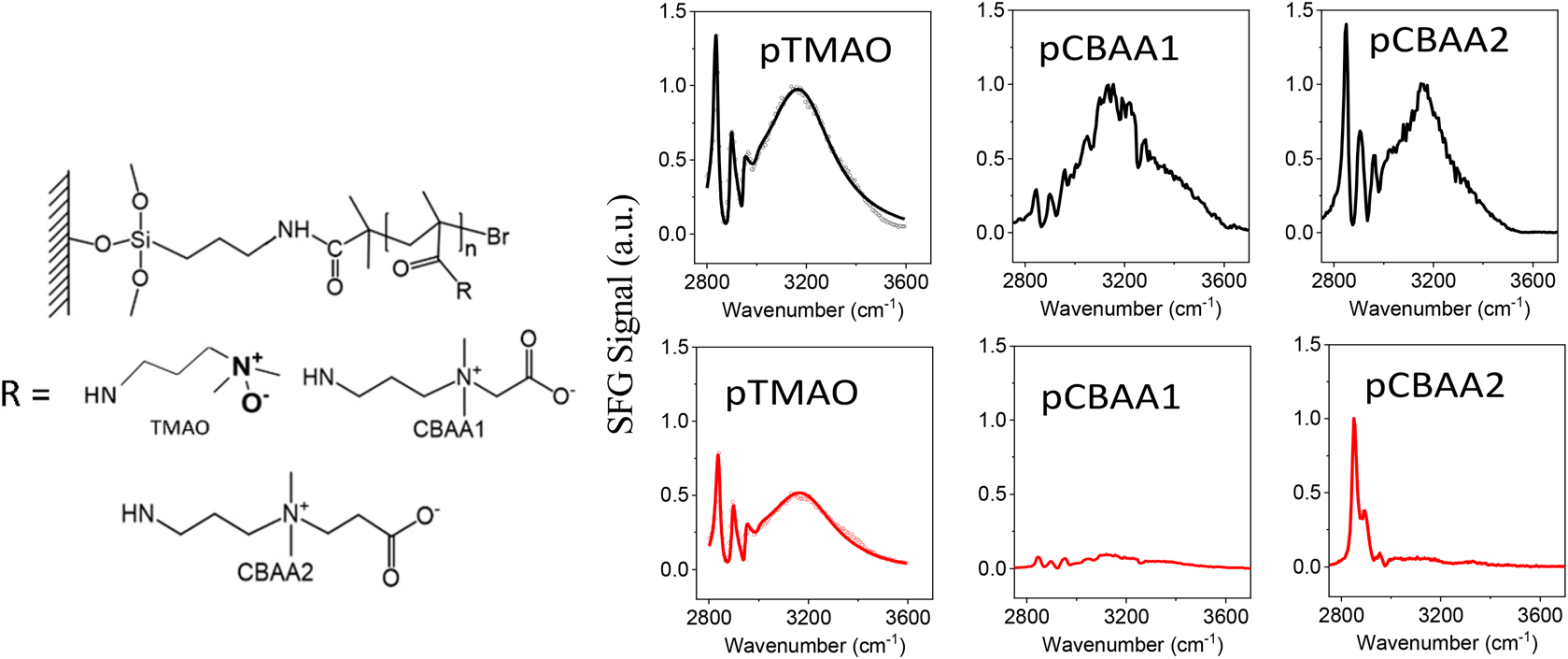
Molecular structures of zwitterionic polymers (left) and the SFG spectra (right) collected from zwitterionic polymers prepared based on TMAO, CBAA1, and CBAA2 in contact with water (colored black) and 0.5 M NaCl solution (colored red). The SFG spectra of pTMAO were adapted from ref. 11 with permission, copyright 2021, American Chemical Society.

SFG vibrational spectroscopy measurements were conducted on an EKSPLA PL 2250 (Vilnius, Lithuania) SFG spectrometer. The details of SFG theories and experiment setup have been previously published^51–55^ and will not be discussed extensively here. Scheme 2 shows the experimental geometry setup of SFG measurements. One visible laser beam (wavelength = 532 nm) overlaps with the other frequency tunable IR beam (wavelength ranges from 2.3 to 10 μm) spatially and temporally on the CaF_2_ prism surface coated by zwitterionic material. The ssp (s-polarized SFG signal output, s-polarized visible beam, p-polarized IR beam) polarization combination was used in this study. The scan range of IR wavenumber is from 2800 to 3600 cm^−1^ with 5 cm^−1^ step size. The SFG spectra, as shown in Fig. 1, were collected when the O–H peak at 3200 cm^−1^ intensity reached equilibrium after water or salt solution was put in contact with zwitterionic material.

### Preparation of polymer brushes

The CBAA polymers (pCBAAs) were grafted onto right-angle SiO_2_ prism surfaces using surface-initiated controlled radical polymerization following a protocol modified from the published methods.^56,57^ Briefly, SiO_2_ prisms were cleaned by immersion in base piranha for 10 min at 80 °C, followed by rinsing with DI water and drying under a stream of air. ATRP (atom transfer radical polymerization) initiator was immobilized onto prism surfaces by soaking clean silica prism in a solution of [11-(2-bromo-2-methyl) propionyloxy] undecyltrichlorosilane (Gelest, 0.05 v% in anhydrous toluene) for 15 min, followed by rinsing with THF and hot water and drying under a stream of nitrogen. Five initiator-coated prisms were placed into a Schlenk flask and deoxygenated *via* four evacuate–refill cycles. 2,2′-bipyridine (624 mg, 4.0 mmol), CBAA (4.3 g for CBAA1; 4.6 g for CBAA2, 20 mmol), methanol (24 mL), and water (6 mL) were added into a separate flask. The mixture was degassed by purging with N_2_ for 30 min before CuBr (286 mg, 2.0 mmol) was added under N_2_ protection. The mixture was then stirred vigorously to become a homogeneous solution and was transferred to the Schlenk flask containing the prisms using a nitrogen-flushed syringe. The reaction was maintained at ambient temperature for 12 h. Afterward, the silica prisms were removed from the solution and washed with methanol and water three times, respectively.

## Results and Discussion

### Experimental studies on hydration and salt resistance

SFG vibrational spectroscopy is a submonolayer surface-interface-sensitive analytical tool.^11,17,51–54,58–62^Fig. 1 shows the SFG spectra collected from zwitterionic polymers based on TMAO, CBAA1, and CBAA2 in contact with water and the 0.5 M NaCl solution. The structures of these three polymers, namely pTMAO, pCBAA1, and pCBAA2 were previously reported.^11,17^ The SFG spectra collected from the water interface contain one dominating peak centered at ∼3200 cm^−1^, which is attributed to the strong hydrogen-bonded interfacial water. This shows that all three zwitterionic materials have strong surface hydration. Fig. 1 also shows that the surface hydration decreased moderately when transferred from water to a 0.5 M NaCl solution, as a relatively strong SFG O–H stretching signal is still present. Differently, when the other two zwitterionic materials (pCBAA1 and pCBAA2) with longer charge separation distances were transferred from water to 0.5 M NaCl solution, the surface hydration decreased significantly, as indicated by the absence of SFG O–H stretching signal in the spectra. The decreased surface hydration observed in pCBAA1 and pCBAA2, when transferred to a 0.5 M NaCl solution, is attributed to the salt screening effect, where the ions in the solution interact strongly with the surfaces of the zwitterionic materials, leading to a substantial reduction in surface hydration. These experimental results^11,17^ demonstrated that the surface hydration and salt resistance for a zwitterionic material are likely to be mediated by the length of spacing between the positive and negative charges in the material. The stronger surface hydration observed on pTMAO resists the salt effect on hydration reduction because of the short distance between the charges. It was also reported in the literature^20^ that for zwitterionic polymers with similar structures, the fouling-resistant activity decreased as the space between the negatively and positively charged groups increased. Therefore, for various pCBAAs, pCBAA1 has the highest fouling-resistant activity, followed by pCBAA2 and pCBAA3. To understand the underlying mechanisms of charge distance-dependent fouling-resistant property of zwitterionic materials, we will next investigate the HB, net charge, and dipole moment of TMAO, CBAA1, CBAA2, and CBAA3.

### AIMD simulations of the hydration of zwitterions

**Fig. 2:**
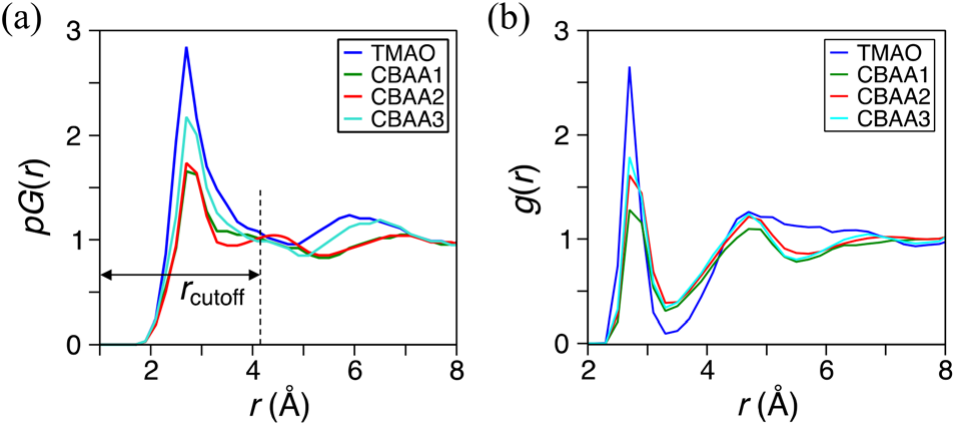
Hydration profiles of zwitterions. (a) Molecular surface hydrations characterized by the proximal radial distribution function *pG*(*r*). (b) Hydrations at the oxygen site with water oxygen characterized by the radial distribution function *g*(*r*). Oxygen atoms in water molecules are used to track the water molecules for both *pG*(*r*) and *g*(*r*). Note: In the *pG*(*r*), the distance *r* is defined as the minimum distance between a water–oxygen atom and any atoms of zwitterions to approximate its distance to the surface of the zwitterion. In contrast, in the *g*(*r*), the distance *r* refers to the distance between water oxygen and the oxygen site of zwitterions.

We applied DFT (density functional theory)-based AIMD simulations^11,21,63^ to study the hydration behavior of zwitterions (TMAO, CBAA1, CBAA2, and CBAA3) taking into account the quantum effects, such as depolarization/polarization and charge transfer in hydration. To quantify the overall and local hydration around the zwitterion surface and the O^−^-site, we calculated the proximal radial distribution function *pG*(*r*) (Fig. 2a)^11,64^ and the radial distribution function *g*(*r*), respectively^65^ (Fig. 2b). Strong hydration peaks at ∼2.7 Å in both *pG*(*r*) and *g*(*r*) were observed for all four zwitterions, indicating a condensed hydration layer surrounding the zwitterion's surface and the local O^−^-site. As validated in the following, the strong hydration peak (*r* ≤ 4.0 Å) in the *pG*(*r*) can be attributed to the presence of both HB and nonHB water molecules, which have strong interactions with a zwitterion. The peak center (∼2.7 Å) of both *pG*(*r*) and *g*(*r*) mainly presents the HB between the water oxygen and the O^−^-site of a zwitterion. Another *pG*(*r*) peak, corresponding to the second hydration shell, is typically visible at ∼6 Å for TMAO and CBAA3. However, in comparison to the first *pG*(*r*) peak, the height of the second one is significantly reduced, indicating that the first hydration shell is the dominant factor in determining the hydration of zwitterionic materials. As such, our following discussions of zwitterionic–water interactions are limited to the first hydration of zwitterions only.

Among four zwitterions, TMAO exhibits the highest peaks in both *pG*(*r*) and *g*(*r*) (Fig. 2 and Table S1 in the ESI†), suggesting its strongest hydration, mainly driven by HB. This finding is highly correlated with salt resistance and fouling-resistant activity of TMAO-containing polymer. CBAA1 and CBAA2 display comparable peak heights and widths in *pG*(*r*), indicating similar hydration on their molecular surfaces (Fig. 2). This observation is consistent with the SFG experimental measurements (Fig. 1). However, the *g*(*r*) peak of CBAA2 around the O^−^-site is relatively higher than that of CBAA1, suggesting a higher density of water molecules around the O^−^-site of CBAA2 compared to that of CBAA1. This implies that the hydration, especially the HB, of the O^−^-site of CBAA1 is relatively weaker, which will be discussed further below. Apparently, here the fouling-resistant activity difference between zwitterionic polymers prepared based on CBAA1 and CBAA2 observed in previous experiments^20^ cannot be explained solely by the factor of hydration. The experiments^20^ showed that CBAA1, with a shorter charge separation distance, exhibited better fouling-resistant activity than CBAA2. It also suggests that hydration strength is insufficient to fully evaluate the fouling-resistant activities of zwitterionic materials. As will be discussed later, net charge and dipole moment are two other additional key factors governing the interactions of zwitterionic materials with biofoulers, *i.e.*, biofouling behaviors.

Fig. 2 also shows that CBAA3 has a higher hydration peak in both *pG*(*r*) and *g*(*r*) than CBAA1 and CBAA2, while the previous experiment^20^ demonstrated that the polymer based on CBAA3 has the lowest fouling-resistant activity among the three CBAA materials. This also indicates that the interfacial hydration of zwitterion is not directly related to the fouling-resistant performance of the CBAA3 material. As will be discussed below, the lowest fouling-resistant activity of CBAA3 is instead due to the longest distance between the positive and negative charges in CBAA3, and the consequential largest dipole moment. Such a long distance between the positive and negative charges also makes the CBAA3 surface more like a locally charged surface rather than a zwitterionic surface, compared to other zwitterionic compounds (TMAO, CBAA1, and CBAA2) with shorter charge separation spacing. Hence, we will present the discussion of CBAA3 at the end of the entire “Results and Discussion” section, rather than in each subsection below.

**Fig. 3:**
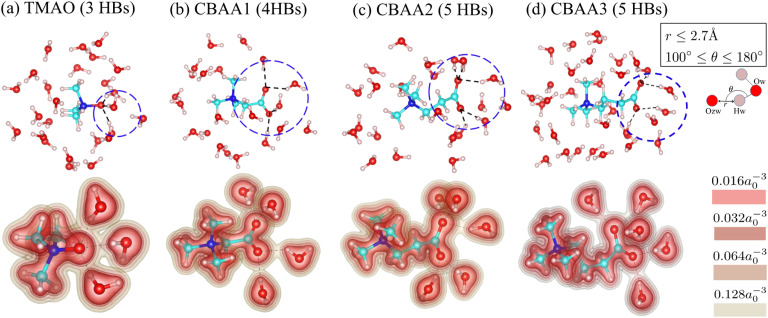
Hydration structures of zwitterions: (a) TMAO; (b) CBAA1; (c) CBAA2; (d) CBAA3. (Top) The first-hydration shells of TMAO and CBAAs using the cutoff distance *r*_cutoff_ = 4 Å according to the *pG*(*r*) profiles (see Fig. 2a). (Bottom) The electron density profiles of zwitterion–HB water complexes. Color bars represent different isolevels set for the charge density plotting. *a*_0_ is the Bohr radius. Black broken lines represent hydrogen bonds between the zwitterion–oxygen and water–hydrogen.

Fig. 3 shows the detailed hydration structures of the four zwitterionic compounds. The existence of the HB was determined based on the geometric criteria and the evaluation of electron charge density overlap^66^ between zwitterion oxygen and water hydrogen (Fig. 3). Evidently, a zwitterion is surrounded by both HB-water and nonHB-water molecules in the first hydration shell (Fig. 3). Most of the HB-water molecules are located around the O^−^-site (*i.e.*, the zwitterionic-head), and the nonHB-water molecules are found around the Me_3_N^+^-site (*i.e.*, the zwitterionic-tail) and the spacer between the O^−^-site and the Me_3_N^+^-site. The number of water molecules that forms HBs with the O^−^-site of zwitterions increases with the charge spacing in zwitterions and the number of O^−^-sites: TMAO has three HBs, CBAA1 has four HBs, and both CBAA2 and CBAA3 have five HBs (Fig. 3). The number of HBs formed by each zwitterion is not related to either the hydration strength or the fouling-resistant activity of zwitterionic materials. However, the differences in HB lengths for different zwitterions in equilibrium are observed: ∼1.55 Å for TMAO, ∼1.75 Å for CBAA1, ∼1.76 Å for CBAA2, and ∼1.71 Å for CBAA3 (Fig. S1 in the ESI†). These variations in HB length should impact the strength of HBs mediated by zwitterionic material–water interactions, which will be discussed below.

**Fig. 4:**
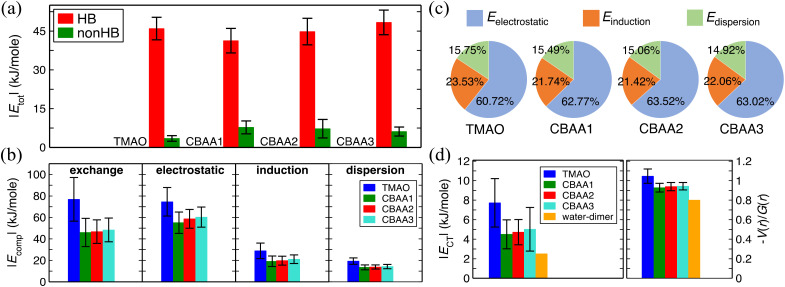
Non-covalent interactions of zwitterions with water. (a) A comparison of HB-water (red) and non-HB-water (green) contributed interactions (|*E*_tot_|) normalized by the number of water molecules participating in the interactions. (b) Four energy components (|*E*_comp_|) of |*E*_tot_|—exchange, electrostatic, dispersion, and induction—of HB-water interactions; only the exchange energy is repulsive, while the others are attractive. |*E*_comp_| values are also normalized by the same number of water molecules as for |*E*_tot_|. (c) The percentage contribution of each attractive energy component in the total attractive interaction. (d) Normalized charge transfer energy |*E*_CT_| and the ratio of potential energy density, *V*(*r*), to kinetic energy density, *G*(*r*), at the bond critical point. |*E*_CT_| is associated with the HB formation and belongs to the induction interaction in Fig. 4b. −*V*(*r*)/*G*(*r*) and |*E*_CT_| are normalized by the number of HBs in the zwitterion–HB-water complex. The water dimmer is shown in Fig. 4d as a reference.

### Enthalpy effect on the hydration of zwitterionic compounds

We quantified the interaction energies of zwitterions with HB-water around the oxygen site(s) of the zwitterion, as well as with the non-HB-bonded water, using the analysis of SAPT^31^ (Fig. 4). The solvated zwitterion structures in equilibrium obtained from the AIMD simulation were used for the analyses. The total interaction energies (*E*_tot_) for both HB-water and non-HB-water are negative (*i.e.*, attractive), indicating that both HB- and nonHB-water contribute to the hydration strength of the zwitterions. However, when comparing the absolute values (|*E*_tot_|) normalized by the number of HB-water or nonHB-water (Fig. 4a), the contribution from each HB-water greatly outweighs the nonHB-water counterpart. This indicates that although the nonHB-water plays a role in the zwitterion–water interactions due to their abundance, the HB-water molecules are crucial to the hydration of zwitterions. The presence of those HB-water generates a physical and energetic barrier around the bare O^−^-site (see Fig. 3), which can effectively prevent interactions with salts and biofoulers. For instance, as demonstrated in our previous atomistic MD simulations^11,12^ that are in consistent with the SFG experiments,^11^ there exists a condensed hydration water layer with an ordered structure on the pTMAO brush surfaces, regardless of the polymer surface packing density. The strong surface hydration is mainly due to the hydrogen bonding between water and TMAO oxygen atoms, which are exposed to the solvent environment.^11,12^ The presence of a strong surface hydration layer creates a significant energy barrier at ∼3 Å above the top of pTMAO brush surfaces,^11,12^ resisting adsorption of proteins of different charges, such as the positively charged lysozyme protein and the negatively charged barnacle cement protein.^12^ The desorption free energy of a lysozyme protein on pTMAO brush surfaces in pure water is only ∼4.0 kT,^12^ which is one order of magnitude lower than that on a *trans*-azobenzene-terminated fouling surface (∼60 kT).^67^ Even for a large-sized barnacle cement protein, its desorption free energy on pTMAO brush surfaces in pure water is only ∼18.5 kT.^12^ The low desorption free energy of proteins also indicates the pTMAO surface's fouling-resistant activity, which results from the overall effect of surface hydration, a near-neutral net charge, and a small dipole moment (both of which will be discussed later).

Fig. 4a shows that the normalized HB energies |*E*_tot_| between a zwitterion and an HB water molecule for TMAO and CBAAs are comparable, with slight variations depending on the zwitterionic spacing: an initial decrease in |*E*_tot_| from TMAO to CBAA1 followed by an increase with the growing zwitterionic spacing. Fig. 4b presents the components of normalized interaction energies (exchange, electrostatic, induction (polarization), and dispersion) between a zwitterion and an HB-water. TMAO exhibits the highest repulsive exchange interaction energy |*E*_exchange_| due to the shortest HB distance (∼1.55 Å; see Fig. S1 in the ESI†). For a similar reason, the absolute values of all attractive components (electrostatic, induction, and dispersion) are also the highest for TMAO. When comparing CBAA1 and CBAA2, the absolute value in each energy category of the interaction energy of CBAA2 is slightly larger. Therefore, the energetic differences between CBAA1 and CBAA2 cannot explain the difference in fouling-resistant activities observed in the experiment.^20^ One important observation from Fig. 4c is that |*E*_electrostatic_| dominates the total attractive interactions for all zwitterions, accounting for around 61–63%. This validates the experimental rule of thumb that the hydration of zwitterions is electrostatically driven.^11^ It is also worth noting that |*E*_induction_| (*i.e.*, the polarization energy) also plays a significant role, as it contributes around 21–24% to the total attractive interaction (Fig. 4c). Moreover, |*E*_induction_| includes the energetic contributions in the HB formation, called charge transfer energy (*E*_CT_), between the zwitterion-oxygen and water–hydrogen. As *E*_CT_ is a measure of HB strength (Fig. 4d),^37,38,68^ the contribution of |*E*_induction_|, although much smaller than |*E*_electrostatic_|, also underlies the strength of an HB.

As the HB strength is critical to the hydration strength of zwitterions, we analyzed its dependence on charge spacing size (Fig. 4d) using the equilibrated solvated conformations of zwitterions from two different perspectives: the charge transfer energy, |*E*_CT_|,^37,38,68^ and the ratio of potential energy density (*V*(*r*)) to kinetic energy density (*G*(*r*)), at the bond critical points between water–hydrogen and zwitterion–oxygen using QTAIM.^40,41^ |*E*_CT_| is an estimation of the extent of charge overlapping between two non-covalently interacting systems, indicating bond stability. The −*V*(*r*)/*G*(*r*) corresponds to the ratio of attractive to repulsive forces between the electrons at the bond critical point and is a measure of bond nature (ionic: −*V*(*r*)/*G*(*r*) < 1; covalent: −*V*(*r*)/*G*(*r*) ≥ 2; partially covalent: otherwise).^40,41^ Thus, the higher the value of these quantities, the stronger an HB, and *vice versa*. Fig. 4d clearly shows that both |*E*_CT_| and −*V*(*r*)/*G*(*r*) values of TMAO are much larger than those of CBAA1 and CBAA2, *i.e.*, TMAO has the strongest HB. This indicates that the stronger HB is likely to mediate the strongest hydration, salt resistance, and nonfouling activity of TMAO polymer. For CBAA1 and CBAA2, both values are similar, suggesting that their difference cannot explain the difference in nonfouling activity of CBAA1 and CBAA2 polymers observed in the previous experiment.^20^

Also, it is worth mentioning that the HB strength between water and TMAO (or CBAAs) is stronger than that of the water dimmer (see |*E*_*CT*_| and −*V*(*r*)/*G*(*r*) values in Fig. 4d). As a result, zwitterions can have stronger hydration around them, as evidenced by the first hydration peaks in *pG*(*r*) peak at ∼2.7 Å (Fig. 2a).

### Conformational entropy effect on the hydration of zwitterionic compounds

To evaluate the structural stability of those zwitterions, we computed their conformational entropy *S* using their solvated structures and the theory of quasi-harmonic approximation.^50^ As shown in Table 1, TMAO has the lowest *S* value (0.121 kJ K^−1^ mol^−1^), indicating the highest structural stability among the four zwitterions, which can be attributed to its shortest spacing between the oppositely charged moieties. In contrast, CBAAs exhibit increasing *S* values with the growing size of the zwitterionic spacer, mainly due to the structural flexibility of the alkane chain spacer and the rotation of the tail and oxygen atoms of the head in CBAAs (see Fig. S2 in the ESI†). The structural stability of zwitterions is closely linked to their hydration strength. TMAO, with the smallest *S*, has the highest surface hydration, whereas all CBAAs have higher conformational entropy, resulting in reduced hydration. Among the CBAAs, CBAA1 has a lower *S* value than the others, making its surface hydration less affected by structural fluctuations. In contrast, the *S* value for CBAA2 is more than 1.6 times larger than that of CBAA1, which can reduce its surface hydration stability significantly.

**Table 1:** Conformational entropy, *S* (kJ K^−1^ mol^−1^)

TMAO	CBAA1	CBAA2	CBAA3
0.121	0.227	0.378	0.384

### Net charge of zwitterionic compounds

To study another key factor, the net charge, which is highly correlated with the fouling resistance of zwitterionic materials, we employed the Bader charge analysis.^40,49^Table 2 shows all four solvated zwitterions have small net positive charges (*q*_net_), which shows the presence of slight charge transfer from a zwitterion to surrounding hydrogen atoms of water molecules. Although the charge transfer is small (*i.e.*, the zwitterions are near charge-neutral), it is critical for the HB. The *q*_net_, when normalized by the number of hydrogen bonds, can be correlated to their HB strength. For example, the ratio between the normalized *q*_net_ of TMAO to that of CBAAs (*q*_net_: TMAO ∼1.7 × CBAA1, ∼1.7 × CBAA2, ∼1.5 × CBAA3) is consistent with the relative comparison of their corresponding |*E*_CT_| values in Fig. 4d. As |*E*_CT_| indicates the covalency of an HB,^68^ the normalized *q*_net_ can thus also be considered as a measure of the underlying HB strength. TMAO has the largest normalized *q*_net_ (*q*_net_ > 0), which means that it has the largest charge transfer per HB, leading to its strongest HB formation and the strongest HB formation and the strongest hydration.

**Table 2:** Net charge *q*_net_ of zwitterionic compounds

	TMAO	CBAA1	CBAA2	CBAA3
Total	0.114 ± 0.018	0.088 ± 0.01	0.11 ± 0.02	0.128 ± 0.02
Normalized by total HBs	0.038 ± 0.006	0.022 ± 0.003	0.022 ± 0.004	0.0256 ± 0.004

**Fig. 5:**
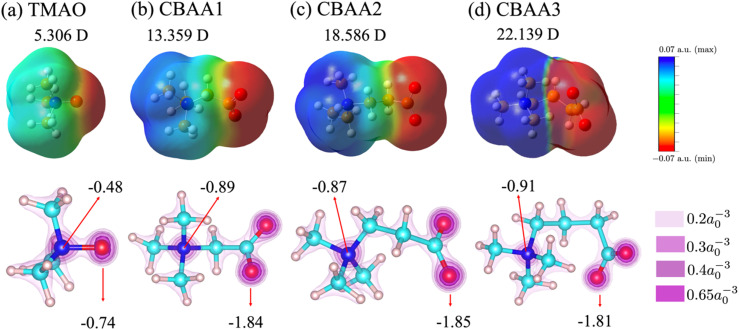
Variation of electron distribution with zwitterionic spacing: (a) TMAO; (b) CBAA1; (c) CBAA2; and (d) CBAA3. (Top) Electrostatic potential surface (EPS) of zwitterions (TMAO, CBAA1, CBAA2, and CBAA3) generated through a constant value (0.0004e a.u.^−3^) of electron density on the van der Waals surface and the electric average dipole moment (in the unit of Debye (*D*)) of each solvated-zwitterion. (Bottom) Electron density profiles of those zwitterions and partial charges of nitrogen and oxygen atoms of zwitterions. Color bars represent different isolevels set for the plotting. *a*_0_ is the Bohr radius. The negative numbers indicate partial charges of N and O atoms. The dipole moment was computed using about 30 solvated structures for each zwitterion at the equilibrium.

The inherent structural advantage of TMAO can result in the highest normalized charge transfer. The lack of spacer between the Me_3_N^+^- and O^−^-sites for TMAO causes the overlap of their charge densities (Fig. 5a). More accumulation of electrons around the oxygen atom is expected due to its higher electronegativity than the nitrogen atom. As these electrons are not directly bound to the positive charge, they can be more easily transferred to the surrounding water. In contrast, CBAAs have a spacer between their Me_3_N^+^ and O^−^-sites, preventing the overlapping of their charges. The electrons of O^−^ in CBAAs are directly and tightly bound to the positive C^+^ (COO^−^; see Table S2 in the ESI†), which makes the charge transfer more difficult compared to TMAO. Hence, the hydrogen bonds in CBAAs are not as strong as those in TMAO.

### Dipole moment of zwitterionic compounds

Dipole moment reveals the nature and strength of electrostatic and dipole–dipole interactions of a zwitterion with biomolecules. Hence, it can be an indicator of the fouling-resistant efficacy of zwitterionic materials. As shown in our previous study of atomistic MD simulation,^69^ the oxygen atoms in CBAAs interact strongly with Na^+^, which is due to their larger absolute values of partial charges and dipole moment (Fig. 5). Similar behavior of CBAAs can be expected with charged biofoulers, as evidenced by the electrostatic potential surfaces depicted in the upper panel of Fig. 5. The region around the zwitterionic head (O^−^-site) becomes increasingly negative (red-colored), while the region around the zwitterionic tail (Me_3_N^+^-site) becomes increasingly positive (blue-colored). As the charge separation distance in a zwitterion increases, the dipole moment also increases, leading to larger electrostatic and dipole–dipole interactions of zwitterionic materials with biofoulers. In the case of CBAAs, increasing the spacing size between charges results in higher |*E*_tot_| (Fig. 4a). However, these increased attractive electrostatic and dipole–dipole interactions with charged biomolecules are not favorable for fouling-resistant properties. In contrast, TMAO has the shortest charge separation distance, which results in the smallest electrostatic potential on the molecular surface and dipole moment (5.306 D) due to the depolarization between its oppositely charged sites. Also, as aforementioned, the direct connection between the Me_3_N^+^ and O^−^-sites in TMAO increases structural stability and charge transfer, leading to the strongest HB strength (partially covalent (1 ≤ −*V*(*r*)/*G*(*r*) < 2) in Fig. 4d). Consequently, TMAO has the most robust hydration. Moreover, the smallest molecular surface electrostatic potential, the smallest atomic partial charge of the TMAO O^−^-site, and the minimum dipole moment all contribute to its strongest fouling-resistant activities. The near-neutral net charge and the small dipole moment of TMAO indicate its weaker electrostatic and dipole–dipole interactions with salts and proteins compared with CBAAs. This is consistent with the observation of the small salt effects on the density and the order structure of surface hydration on the pTMAO brush surface (see pTMAO in Fig. 1), as well as the protein desorption free energy.^11,12^

Although TMAO has the smallest dipole moment (5.306 D) compared to CBAAs, it is still larger than that of water (1.86 D). Such a larger dipole maximizes the interaction of TMAO with water molecules, resulting in stronger HB and hydration. However, it also enhances the dipole–dipole interactions of zwitterionic materials with biofoulers. As shown in our previous studies,^11,12^ the robust hydration of TMAO can minimize both the protein desorption energy. The value of TMAO dipole moment (5.306 D) does not operate as large enough for TMAO to let the dipole–dipole interactions take over its hydration strength.

To gain more insight into the dipole moment of zwitterions, we analyzed their partial charges of O and N atoms (see the lower panel of Fig. 5). The partial charge of oxygen changes substantially from approximately −1.81 to −0.74 when the –(CH_2_)_*n*_– spacer is removed. Similarly, a notable partial charge variation from approximately −0.91 to −0.48 was also observed on nitrogen atoms, illustrating a remarkable depolarization effect due to the removal of the hydrophobic spacer. This strong depolarization of the zwitterionic groups in TMAO arises from the absence of the bridging carbon atoms, which are electropositive and thus would otherwise polarize the electronegative oxygen and nitrogen atoms.

### Discussion on CBAA3

Previous research^20^ reported that CBAA3 polymer has lower fouling-resistant activity compared to CBAA1 and CBAA2 polymers. The results presented above show that many properties of CBAA3 in hydration behavior and zwitterionic–water interaction energies are intermediate between TMAO and CBAA1 or CBAA2. If such properties determined the fouling-resistant activity, CBAA3 would exhibit better fouling-resistant activity than CBAA1 and CBAA2, which was not observed experimentally.^20^ Due to such a large distance between the negative and positive charges, CBAA3 loses some zwitterionic characters while possessing some behaviors of a charged compound, compared to CBAA1 and CBAA2. Our simulation shows that CBAA3 has the largest dipole moment, which can lead to an increase in its electrostatic and dipole–dipole interactions with biofoulers. Therefore, its fouling-resistant activity^20^ could not be explained by its hydration behavior. However, these properties are useful for understanding the activity of zwitterionic materials in pure and salt water. We thus included the results of CBAA3 in the data presented in this research to provide a more complete picture for studying the effects of distance between different charges on the properties of zwitterionic materials.

## Conclusions

In this work, we studied the hydration behaviors of zwitterionic materials (TMAO and carboxybetaines) of different charge-separation distances, using quantum simulations and nonlinear vibrational spectroscopy. Our findings on the hydration properties (HB, net charge, and dipole moment) of these materials were utilized to interpret their protein-resistant functionalities, which were measured in the experiments.^11,20^ This allowed us to gain an in-depth understanding of the underlying molecular mechanisms of fouling resistance of zwitterionic materials.

Our work demonstrates the importance of hydration behaviors of zwitterionic materials in exhibiting fouling-resistant activities. Specifically, the interplay among hydrogen bonding, net charge, and dipole moment determines the fouling resistance. Our study revealed that hydrogen bonding plays a crucial role in the hydration of zwitterionic materials. In zwitterionic compounds like TMAO, the closer spacing between the oppositely-charged moieties results in higher water density in the hydration layer, stronger interactions with HB-water, and a more stable HB structure, providing a protective hydration barrier against the biomolecular attachment. Furthermore, the intrinsic near-neutral charge and reduced dipole moment of a zwitterion, which results from the shortened charge separation distance, can decrease its attractive electrostatic interactions and dipole–dipole interactions with biofoulers, thereby enhancing the fouling-resistant activities.

Although there was no discernible difference in overall hydration on the entire molecular surface between CBAA1 and CBAA2 compounds, a difference is observed in the strength of hydrogen bonding of their O^−^-sites. Compared to CBAA2, CBAA1 has a relatively weaker interaction with HB-water but larger structural stability in hydrogen bonding (*i.e.*, the conformational entropy effect). CBAA1 also has a smaller dipole moment, which can enhance its fouling-resistant activities by reducing its electrostatic and dipole–dipole interactions with biofoulers. As the spacing is increased, such as in CBAA3, it can have strong hydration, but its fouling-resistant activity is lower compared to other zwitterionic compounds (CBAA1 and CBAA2) due to its large dipole moment. This research demonstrates the importance of having a short distance between charges in designing new zwitterionic materials with excellent fouling-resistant properties.

SFG results on different salt-resistant behaviors of various zwitterionic polymer materials demonstrate different surface hydrations of these zwitterionic materials. Our *ab initio* calculations did not provide a direct interpretation of the salt resistance of zwitterionic molecules. To elucidate the salt resistance mechanism of TMAO, future simulation research, including salt molecules in the zwitterionic molecule–water system, should be performed, which is beyond the scope of this research.

While our study focused on the zwitterionic materials of CBAA1, CBAA2, CBAA3, and TMAO, these findings provide an in-depth understanding of their hydration behavior and interpret the fouling-resistant mechanism, which are critical to developing nonfouling materials in the future.

## Data availability

Data supporting the findings of this work are available within the paper and its ESI.† Additional data related to this paper may be requested from the author tao.wei@howard.edu on reasonable request.

## Author contributions

P. S., S. J., Z. C. and T. W. designed research. P. S. performed AIMD, DFT, and SAPT calculations. M. S. J. S. performed MD calculations. D. L. synthesized materials and prepared samples. T. L. and G. W. performed SFG experiments. P. S., T. L., D. L., H. C., S. J., Z. C, and T. W. analyzed data. T. L., D. L., and H. C. contributed to writing. P. S., S. J., Z. C., and T. W. wrote and edited the manuscript. S. J., Z. C. and T. W. supervised the project.

## Conflicts of interest

The authors declare no conflict of interest.

## Supplementary Material

SC-014-D3SC01977B-s001
