# Supplementary material for: Hydration behaviors of nonfouling zwitterionic materials
Source: Chem Sci. 2023 Jun 6;14(27):7500–11. doi: 10.1039/d3sc01977b (PMC10337769; doi:10.1039/d3sc01977b)
Supplement: SC-014-D3SC01977B-s001 [file SC-014-D3SC01977B-s001.pdf]

Electronic Supplementary Material (ESI) for Chemical Science.
This journal is © The Royal Society of Chemistry 2023

Electronic Supplementary Material (ESI) for Chemical Science.
This journal is © The Royal Society of Chemistry 2023

Supplementary Information

Hydration Behaviors of Nonfouling Zwitterionic Materials

Pranab Sarker[a], Tieyi Lu[b], Di Liu[c], Guangyao Wu[b], Hanning Chen[4], Md Symon Jahan Sajib[1], Shaoyi Jiang[3]*, Zhan Chen[2]*, Tao Wei[a]*

[a]Department of Chemical Engineering, Howard University, Washington, D.C., United States

[b]Department of Chemistry, University of Michigan, Ann Arbor, Michigan, United States

[c]Meinig School of Biomedical Engineering, Cornell University, New York, United States

[d]Texas Advanced Computing Center, The University of Texas at Austin, Austin, Texas, United States

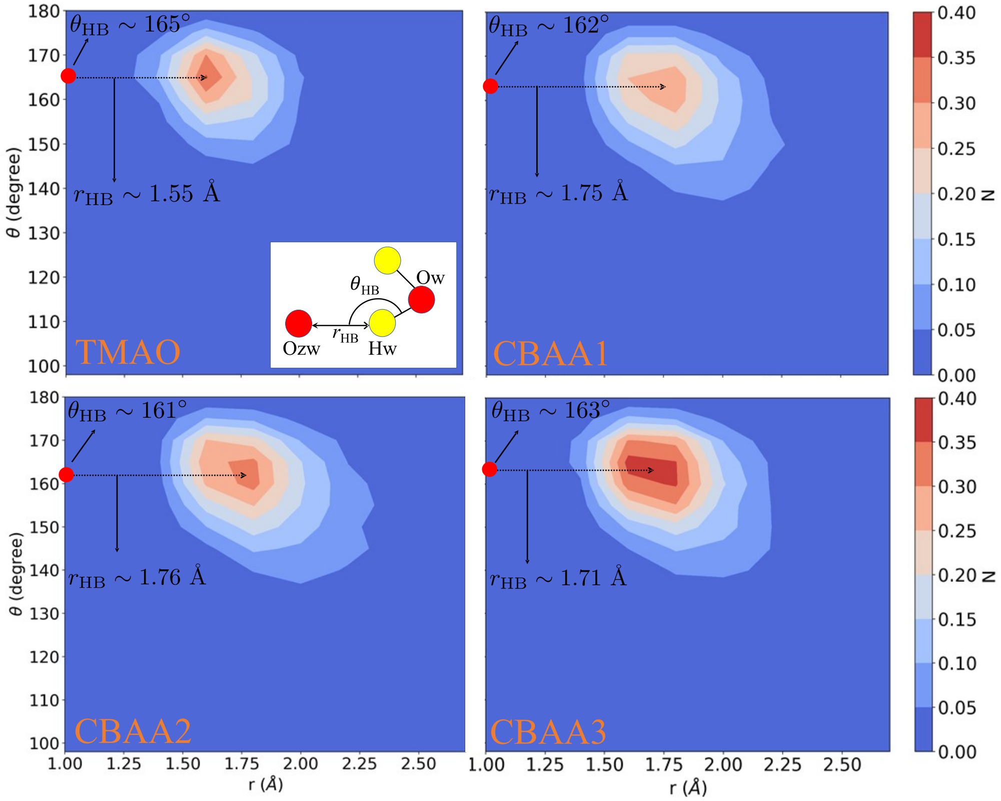

Figure S1. Time-averaged distribution (*N*) of H-bond between water hydrogen (H_w) and zwitterionic oxygen (O_{zw}). The center of the hotspot in each plot is taken as the equilibrium bond distance and angle for TMAO and CBAAs.

Table S1. Comparison of *pG*(*r*) and *g*(*r*) peak heights

	pG(*r*) peak height	*g*(*r*) peak height
TMAO	2.85	2.64
CBAA1	1.66	1.25
CBAA2	1.74	1.61
CBAA3	2.18	1.8

Note: The data were taken from Figure 2.

Geometric criteria for an H-bond (HB)

In the present work, we only consider the HB between zwitterionic oxygen(s) and a water molecule. The distance (r_{HB}) between the zwitterionic oxygen and water-hydrogen and the angle (θ_{HB}) between zwitterionic oxygen, water-hydrogen, and water-oxygen ($\theta_{HB} = \angle O_{zwitterion}H_{water}O_{water}$) (see **Figure S1**) are used as geometric criteria for an HB to be counted. As the extent of r_{HB} for a weaker H-bond can be more than 3.0 A (1), we choose a stricter criterion for it, $r_{HB} \leq 2.7$ A, as the stronger HBs are critical to stronger hydration, with $100^{\circ} \leq \theta_{HB} \leq 180^{\circ}$. $r_{HB} \leq 2.7$ A corresponds to the position of *pG*(*r*) peaks (maximum hydration strength for a zwitterion) in **Figure 2a**. Based on these criteria, we obtained the time-averaged distribution of HB (normalized by the total number of configurations used for the analyses over 120 ps (200 ps) for TMAO (CBAAs). In the case of CBAAs, if the same water hydrogen makes an HB with both oxygens of CBAAs, only one H-bond is counted.

Origin of CBAA's high-configuration entropy (*S*)

Figure S2a illustrates the root-mean-square-displacements (RMSDs) of TMAO and CBAAs. It is evident from the figure that the RMSDs for finite-spacing CBAAs can be very high (compared to the zero-spacing TMAO), giving rise to their high *S*. As shown in **Figure S2b**, the high-RMSD of each CBAA arises from the rotation of either the tail or head or both, with respect to a reference configuration (e.g., the orange structures in **Figure S2b**). The RMSDs and overlaps between the reference and transition structures are generated using VMD (3). In the case of CBAA1, the high-RMSD values originate primarily either by the rotation of the tail or head. For convenience, we consider the rotation of the heads (O1 and O2) only, as the overlapping between the reference and transitioning structures provides the best fit with the tails. While comparing with the low-RMSD reference structure (orange-CBAA1), only the head rotates, undergoing a full 180° swing (see the alternating transition in snapshots from 1-4 for CBAA1 in Fig. S4b). As a result, its *S* (0.227 kJ/K-mole) is lower than other CBAAs. In contrast, both the tail and head of CBAA2 rotate, resulting in a higher value of *S* (0.378 kJ/K-mole), although, unlike CBAA1, its head (O1 and O2) does not undergo a full swing. In the case of CBAA3, both the tail and head rotate with the full swing (see snapshots 1 and 2 for CBAA3 in **Figure S2b**). As such, the *S* is the highest for CBAA3.

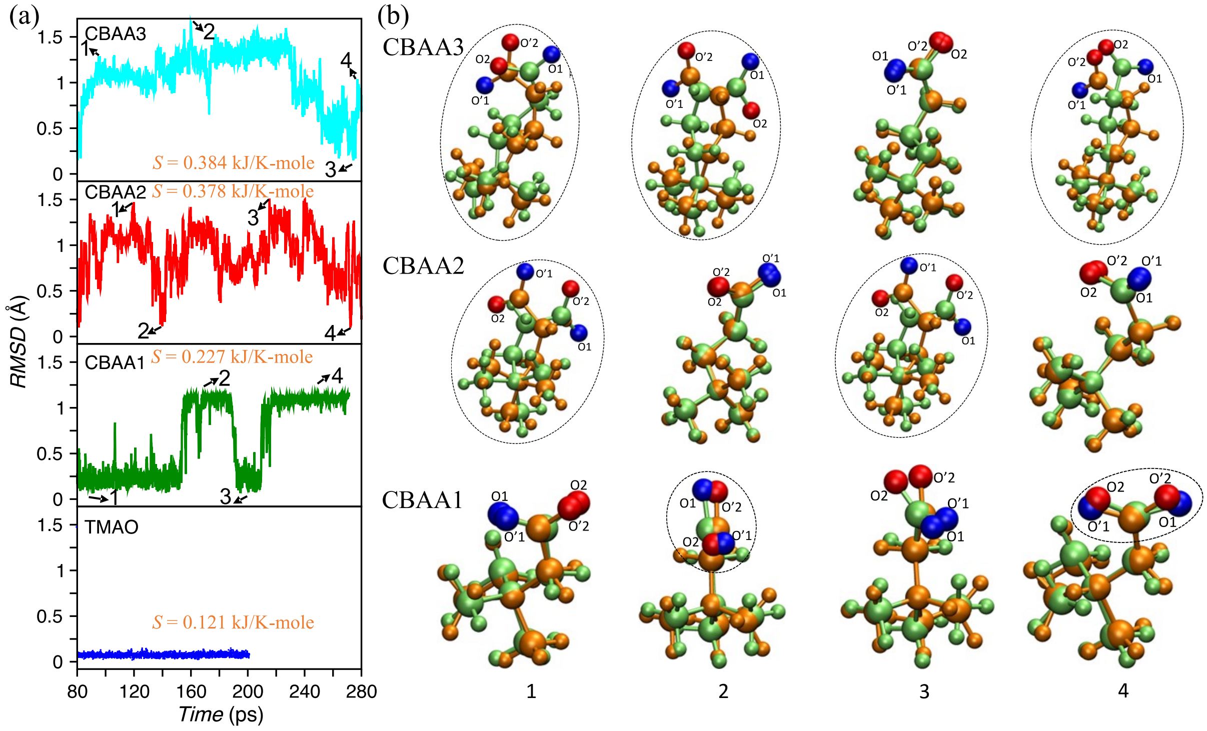

Figure S2. Structural fluctuations of TMAO and CBAAs. (a) The root-mean-square-displacement (RMSD) and the configurational entropy (*S*; calculated via Schlitter formula (2) see Methods). (b) Four overlapped configurations of each CBAA (green) with the respective reference structure (orange) taken at 80th ps. Blue-$O1'$ ($O1$) and red-$O2'$ ($O2$) are the oxygen atoms in the reference (transition) structures of CBAAs.

Table S2. Bader charges (4,5) of C in the COO^- of solvated-CBAAs.

	CBAA1	CBAA2	CBAA3
C (COO^-)	2.93 ± 0.18	2.88 ± 0.07	2.75 ± 0.12

Estimation of partial charges of TMAO and CBAAs for MD simulations

The initial configurations of TMAO and CBAAs were pre-relaxed with MD simulations. The partial charges needed for carrying out MD simulations were obtained following the protocols outlined in our previous work (6) within the restrained ESP or RESP-AMBER fitting procedure (7). The Hartree-Fock *ab initio* method with a 6-31* basis set, as suggested in the AMBER protocol, was employed to estimate the electrostatic potentials (ESP) of the gas-phase zwitterions. Those ESP data were then fed into RESP-AMBER fitting procedure to calculate the partial charges. Before calculating the ESPs of all zwitterions, their geometries were optimized within the DFT framework. The B3YLP functional with 6-311G (2d, p) basis set and D3 version of Grimme's dispersion with Becke-Johnson damping (GD3BJ) (8) were used. Gaussian16 package (9) was utilized for both optimization and ESP calculation.

Reference:

1. Gilli, G.; Gilli, P. The nature of the hydrogen bond: outline of a comprehensive hydrogen bond theory (Oxford university press), **2009**.

2. Schlitter, J. Estimation of absolute and relative entropies of macromolecules using the covariance matrix. *Chem. Phys. Lett.* **1993**, 215, 617–621.

3. Humphrey, W.; Dalke, A.; Schulten, K. VMD: Visual molecular dynamics. *J. Mol. Graph.* **1996,** 14, 33–38.

4. Bader, R. F. W.; Nguyen-Dang, T. T. "Quantum Theory of Atoms in Molecules–Dalton Revisited" *Adv. Quantum Chem.* **1981**, 14, 63–124.

5. Henkelman, G.; Arnaldsson, A.; Jónsson, H. A fast and robust algorithm for Bader decomposition of charge density. *Comput. Mater. Sci.* 2006, *36*, 354–360.

6. Huang, H.; Zhang, C.; Crisci, R.; Lu, T.; Hung, H.-C.; Sajib, M.S.J.; Sarker, P.; Ma, J.; Wei, T.; Jiang, S.; Chen, Z. Strong surface hydration and salt resistant mechanism of a new nonfouling zwitterionic polymer based on protein stabilizer TMAO. *J. Am. Chem. Soc.* **2021**, *143*, 16786-16795.

7. Bayly, C.I.; Cieplak, P.; Cornell, W.; Kollman, P.A. A well-behaved electrostatic potential based method using charge restraints for deriving atomic charges: the RESP model. *J. Phys. Chem.* **1993**, *97*, 10269-10280.

8. Grimme, S.; Ehrlich, S.; Goerigk, L. "Effect of the damping function in dispersion corrected density functional theory," *J. Comp. Chem.* 2011, *32*, 1456-65.

9. Frisch, M. J.; Trucks, G. W.; Schlegel, H. B.; Scuseria, G. E.; Robb, M. A.; Cheeseman, J. R.; Scalmani, G.; Barone, V.; Petersson, G. A.; Nakatsuji, H.; Li, X.; Caricato, M.; Marenich, A. V.; Bloino, J.; Janesko, B. G.; Gomperts, R.; Mennucci, B.; Hratchian, H. P.; Ortiz, J. V.; Izmaylov, A. F.; Sonnenberg, J. L.; Williams-Young, D.; Ding, F.; Lipparini, F.; Egidi, F.; Goings, J.; Peng, B.; Petrone, A.; Henderson, T.; Ranasinghe, D.; Zakrzewski, V. G.; Gao, J.; Rega, N.; Zheng, G.; Liang, W.; Hada, M.; Ehara, M.; Toyota, K.; Fukuda, R.; Hasegawa, J.; Ishida, M.; Nakajima, T.; Honda, Y.; Kitao, O.; Nakai, H.; Vreven, T.; Throssell, K.; Montgomery, J. A., Jr.; Peralta, J. E.; Ogliaro, F.; Bearpark, M. J.; Heyd, J. J.; Brothers, E. N.; Kudin, K. N.; Staroverov, V. N.; Keith, T. A.; Kobayashi, R.; Normand, J.; Raghavachari, K.; Rendell, A. P.; Burant, J. C.; Iyengar, S. S.; Tomasi, J.; Cossi, M.; Millam, J. M.; Klene, M.; Adamo, C.; Cammi, R.; Ochterski, J. W.; Martin, R. L.; Morokuma, K.; Farkas, O.; Foresman, J. B.; Fox, D. J. Gaussian 16, Rev.A.03, Gaussian, Inc. Wallingford CT, **2016**.
